# FUNCTIONAL OUTCOMES OF ACCELERATED REHABILITATION PROTOCOL FOR ANTERIOR CRUCIATE LIGAMENT RECONSTRUCTION IN AMATEUR ATHLETES: A RANDOMIZED CLINICAL TRIAL

**DOI:** 10.2340/jrm.v56.12296

**Published:** 2024-02-22

**Authors:** Omar M. ELABD, Ahmad H. ALGHADIR, Abeer R. IBRAHIM, Shahnaz HASAN, Moattar R. RIZVI, Ankita SHARMA, Amir IQBAL, Aliaa M. ELABD

**Affiliations:** 1 Department of Orthopedics and its Surgeries, Faculty of Physical Therapy, Delta University for Science and Technology, Gamasa, Egypt; 2 Department of Rehabilitation Sciences, College of Applied Medical Sciences, King Saud University, Riyadh 11433, Saudi Arabia; 3 Basic Science Department, Faculty of Physical Therapy, Cairo University, Cairo, Egypt; 4 Department of Physiotherapy and Health Rehabilitation, College of Applied Medical Sciences, Majmaah University, Al-Majmaah, Saudi Arabia; 5 Department of Physiotherapy, School of Allied Health Sciences, Manav Rachna International Institute and Studies (MRIIRS), Faridabad, India; 6 Basic Science Department, Faculty of Physical Therapy, Benha University, Al-Qalyubia, Egypt; 7 Department of Physical Therapy, Aqaba University of Technology, Aqaba, Jordan; 8 Department of Physiotherapy, College of Applied Medical Sciences, Umm Al-Qura University, Makkah, Saudi Arabia

**Keywords:** rehabilitation protocol, anterior cruciate ligament reconstruction, knee pain, functional outcomes

## Abstract

**Background:**

Anterior cruciate ligament (ACL) rupture is the most common knee injury among athletes, and can result in long-term complications and career-ending conditions for sportspeople. There is no consensus in the literature on the effectiveness of rehabilitation after ACL reconstruction, or the best protocol to follow for functional outcome improvement.

**Objective:**

To determine the impact of an accelerated rehabilitation protocol on knee functional outcomes in amateur athletes with anterior cruciate ligament reconstruction (ACLR).

**Design:**

Two-arm, parallel-group randomized comparative design.

**Patients:**

A total of 100 amateur male athletes (mean age 22.01 ± 1.79 years) with ACLR were randomly divided into experimental and control groups (*n* = 50/group).

**Methods:**

An accelerated rehabilitation protocol and a conventional rehabilitation protocol were used for the experimental group. In contrast, only the conventional rehabilitation protocol was used for the control group. The rehabilitation was delivered in 5 weekly sessions for 22 weeks. The primary outcome measure, knee pain, was measured using a visual analogue scale (VAS). Extensive test batteries, for hop tests, Knee Injury and Osteoarthritis Outcome Score (KOOS), and knee effusion, were measured, aiming to add more objective criteria to determine functional performance.

**Results:**

Both groups (*n* = 50/group) were well-matched (*p* = 0.816), with insignificant differences in their demographic characteristics (*p* > 0.05). A multivariate analysis of variance (MANOVA) test showed no significant difference between the 2 groups (*p* = 0.781) at baseline. A 2-way MANOVA (2 × 2 MANOVA) of within- and between-group variations indicated overall significant treatment, time, and treatment × time interaction effects (*p* < 0.001) in favour of the accelerated rehabilitation group.

**Conclusion:**

The accelerated rehabilitation protocol was more effective in improving functional outcomes than a conventional rehabilitation protocol in amateur athletes with ACLR.

LAY ABSTRACT

Anterior cruciate ligament rupture is the most common knee injury among athletes, and can result in long-term complications and career-ending conditions. There is no consensus in the literature on the effectiveness of treatment or the best protocol to follow for knee pain and functional outcome improvement in such injuries. There are a range of rehabilitation protocols. This study examined the effectiveness of an accelerated rehabilitation protocol in amateur male athletes with anterior cruciate ligament reconstruction. The protocol was followed for 5 sessions per week for 22 weeks, with the aim of improving knee pain and functional outcomes so that the patients might regain mobility and muscle function and return to sports participation. The results showed that an accelerated rehabilitation protocol may improve knee pain and functional outcomes in amateur athletes with anterior cruciate ligament reconstruction more effectively than a conventional rehabilitation protocol. In conclusion, physiotherapists should include an accelerated rehabilitation protocol for more rapid recovery and improved functional outcomes when treating amateur athletes with anterior cruciate ligament reconstruction.

Knee injuries are frequent among athletes, with football players at exceptionally high risk. Of these injuries, rupture of the anterior cruciate ligament (ACL), which plays a crucial role in neuromuscular control, is the most common, and can result in long-term complications and career-ending conditions (1–4). ACL reconstruction (ACLR) is a cost-effective treatment strategy for most athletic ACL tears, aiming to restore normal anatomy and biomechanics of the injured knee in order to reduce the incidence of subsequent injuries. Rehabilitation after ACLR is crucial in order to regain mobility and muscle function and return to sports participation. The type of rehabilitation protocol is one of the most critical factors that affects outcome after ACLR (5–7). Several post-ACLR rehabilitation protocols have been proposed to improve muscle strength and knee stability through muscle-strengthening exercises and exercises to improve joint proprioception. The proposed protocols are based mainly on the graft’s biological tissue healing and remodelling time-frames (7–9).

Conventional rehabilitation protocols emphasize pain reduction, full passive knee extension, quadriceps strength training, immediate motion, immediate partial weight-bearing with correct gait pattern without any complications, and functional exercises (8). Cryo-therapy can be used in the first postoperative week to reduce pain (10). In addition, electrostimulation can help to re-educate voluntary contraction of the quadriceps muscles (11).

The accelerated rehabilitation protocol following modified to Hamstrings (HT) ACLR is based on evidence regarding complications, such as graft elongation or rupture, and the advantages mentioned in the literature. The suggested accelerated protocol has been reported to be non-harmful (16). Studies favouring an accelerated rehabilitation programme suggest better graft healing, an increased range of movement, and a reduced risk of graft laxity with an early return to sport (8, 12, 13). However, there is no consensus in the literature on the effectiveness of treatment or the best type of protocol to be followed (8, 14), as 35–45% of injured athletes may not return to perform at their premorbid levels (14, 16), and recurrent ACL injuries after primary ACLR can be devastating and carry a risk of long-term functional deficits with a rate ranging from 1% to 11% (17). Also, objective criteria are lacking to determine functional performance (18, 19).

Therefore, this study aimed to fill a gap in the current literature by determining the impact of an accelerated rehabilitation protocol on knee pain and functional outcomes in amateur athletes with ACLR. The study findings will be valuable to healthcare providers, coaches, and athletes when deciding on the most appropriate rehabilitation protocol. This study aims to provide insights into the optimal timing and intensity of rehabilitation exercises for patients with ACLR, which can inform the development of more effective rehabilitation protocols for this patient population. The study findings could have important implications for athlete safety and long-term outcomes, which could improve patient care and reduce healthcare costs by returning athletes to sport more safely, reducing the risk of re-injury, prolonged rehabilitation, or the need for additional medical interventions. By evaluating the functional outcomes of the accelerated rehabilitation protocol for ACLR in amateur athletes, the study can help identify potential limitations or areas for improvement in the protocol, ultimately leading to better patient outcomes.

## METHODS

In accordance with the Declaration of Helsinki 1964 and its later amendments, a clinical trial was conducted from 2021 to 2023 at physical therapy outpatient clinic of the Delta University, Gamasa, Egypt. The trial was randomized, controlled, and single-blinded. It received approval from Cairo University’s Faculty of Physical Therapy Research Ethics Committee (approval number P.T.REC/012/004421) and was registered with ClinicalTrials.gov under the protocol registration and results system (identifier NCT05716529).

### Sample size determination

The G*Power program (Version 3.1, Kiel, Germany) was used to calculate the anticipated desired sample size. For pain as the primary outcome measure, and an alpha level of 0.05, a desired power of 80%, a 2-tailed *t*-test, a 1:1 allocation ratio, and a 0.52 effect size of the unpublished pilot study, 94 patients were the estimated sample, with 47 patients in each group. To account for the almost 25% expected dropout rate, it was estimated to be necessary to include a total of 125 participants in the study.

### Participants

Amateur male football players who underwent ACLR were invited to participate in this study by their orthopaedic surgeon. They were screened for their eligibility criteria by an external assessor. The inclusion criteria consisted of primary ACLR surgery with a hamstring graft, 18–35 years old, minimal knee effusion, full extension, good patellofemoral mobility, and active control of the quadriceps. The exclusion criteria consisted of having ACL revision surgery, ACLR with any graft other than a hamstring graft, associated medial or lateral ligamentous injuries, a previous meniscectomy or meniscal repair, or cartilage damage (20, 21). Following a detailed explanation of the methods, every patient was required to provide written informed consent to participate in the research.

### Randomization and allocation

A random number table was generated using a computer before data collection began to ensure concealed allocation and simple randomization. The researcher who created the table was not involved in recruiting or treating patients. The assigned intervention groups were written on sequentially numbered index cards, which were folded and put in sealed, opaque envelopes. Blinded to the baseline examination findings, a second therapist opened the envelopes and administered the treatment based on the group assignment. The control group (group 1) received a conventional protocol, while the experimental group (group 2) received an accelerated protocol during their initial examination.

### Outcome measures

The primary outcome measure was pain intensity, measured on a visual analogue scale (VAS). The VAS is a valid and reliable method to measure symptoms in a continuous manner by measuring the distance from the lefthand end of the line to the point marked by the patient, reflecting their pain (22, 23). Extensive test batteries for hop tests, Knee injury and Osteoarthritis Outcome Score (KOOS), and knee effusion were measured, aiming to add more objective criteria to determine functional performance.

The modified single-leg hop test battery is a reliable and valid measure of limb symmetry index (LSI), with an 82% sensitivity rate. The test battery consists of 5 steps: a vertical jump, a hop for distance, a drop jump followed by a double hop for distance, a square hop, and a side hop. Patients were familiarized with the testing facility and completed a standardized warm-up before the test, which included stationary cycling, squats, toe rises, and warm-up jumps. Verbal encouragement was used, and 3–5 practice trials were followed by 3 maximum approved trials for the vertical jump, hop for distance, and drop jump, followed by a double hop for distance. If subjects improved their hop performance, additional hops were performed until no increase was observed. The side hop and square hop were tested once, with 3 min rest between each hop test. The best trial for each leg in each test was used for data analysis, and the result of each item of the hop test battery of the injured limb was divided by the corresponding score of the uninjured limb and multiplied by 100. The same assessor supervised the tests, and athletic footwear was standardized (24–26).

The KOOS is a widely used self-administered questionnaire that assesses patient-reported outcomes in individuals with a knee injury. It consists of 5 subscales: pain, other symptoms, function in daily living, function in sport and recreation, and knee-related quality of life (QoL). Each subscale contains a set of items that assess various aspects of knee-related problems, with a 0–100 scale, where higher scores indicate better outcomes (0 = extreme knee problems, 100 = no knee problem) (27). The KOOS is widely used in clinical and research settings and has been found to be a reliable, valid, and responsive measure for assessing the effectiveness of treatments and interventions to improve knee function and QoL in individuals with a knee injury or osteoarthritis (28, 29).

The knee effusion grading scale is a reliable, valid, and commonly used tool in research and clinical settings to assess the severity of knee effusion and diagnose and monitor various knee conditions, such as ligament injuries. This grading scale quantifies knee effusion severity from 0 to grade 3, with each grade corresponding to an increasing level of effusion severity. Grade 0 indicates no effusion, while grade 1 indicates a small amount of fluid within the joint. Grade 2 indicates a moderate amount of effusion with visible joint line blurring, and grade 3 indicates a large amount of effusion with marked joint line blurring and visible distension of the suprapatellar pouch (30).

### Treatment protocols

Both groups of patients underwent 5 treatment sessions per week for a period of 22 weeks. They were given thorough instructions on performing their exercises and were permitted to do so independently after completing 3 supervised trials with the same physical therapist. As the patients were able to perform more repetitions than the specified amount, the training loads were gradually increased by 2–10%. The exercises were performed slowly to maintain control of the movements and there was a rest period of 2–5 min between each set. During weight-training, patients were allowed to experience pain, but if it exceeded 5 on a 1–10 VAS, the load, range of motion, or both were reduced (31).

The control group patients were given a conventional physical therapy programme (Appendix S1) incorporating many of the ACLR recommendations, such as pain reduction, cryotherapy, full passive knee extension, electrostimulation, quadriceps strength training, immediate motion, immediate partial weight-bearing, and functional exercises (32, 33).

Patients in the experimental group received an accelerated rehabilitation protocol (Appendix S2) that was divided into 4 phases over 22 weeks: the immediate postoperative phase (week 1), the early rehabilitation phase (weeks 2–8), the advanced activity phase (weeks 9–16), and the return to activity phase (weeks 16–22). The protocol aimed to achieve full weight-bearing, passive knee range of motion, reduced post-operative swelling, pain, and inflammation, improved proprioception, complete passive knee extension, gradual strengthening of the hamstring and quadriceps muscles, optimal strength in the lower limbs, better neuromuscular control, and increased strength in the knee-stabilizing muscles. In addition, patients underwent proprioception exercises, stability and balance exercises, and sports-specific exercises (8, 34).

### Data analysis

The reported data were analysed using IBM Statistical Package for Social Sciences (SPSS) "for Windows, Version 26. Armonk, NY: IBM Corp. with an intention-to-treat analysis. When post-intervention data for 7 patients (4 from the control group; 3 from the experimental group) were missing, they were asked for an outcomes examination just after the last performed session before their withdrawal, and these scores were used for statistical analysis (35). The normality of the recorded data for all variables, except knee effusion, was assessed using the Shapiro–Wilk test. The studied variables did not significantly deviate from a normal distribution. Descriptive statistics, such as mean and standard deviation, were calculated and presented in a table for both groups.

A mixed-design multivariate analysis of variance (2 × 2 MANOVA) was conducted to examine within-group and between-group differences. The model included 1 between-group variable with 2 levels (control or experimental treatment), 1 repeated measure variable with 2 levels (pre- or post-intervention assessment time), and an interaction factor (treatment × time). Non-parametric statistics were employed for knee effusion, which was scored ordinally. Mann–Whitney *U* test was used to compare between-group differences, while within-group comparisons were conducted using the Wilcoxon sign-rank test. All measurement tests were based on a 95% confidence interval (95% CI) and a significance level of *p* ≤ 0.05.

## RESULTS

**Fig. 1:**
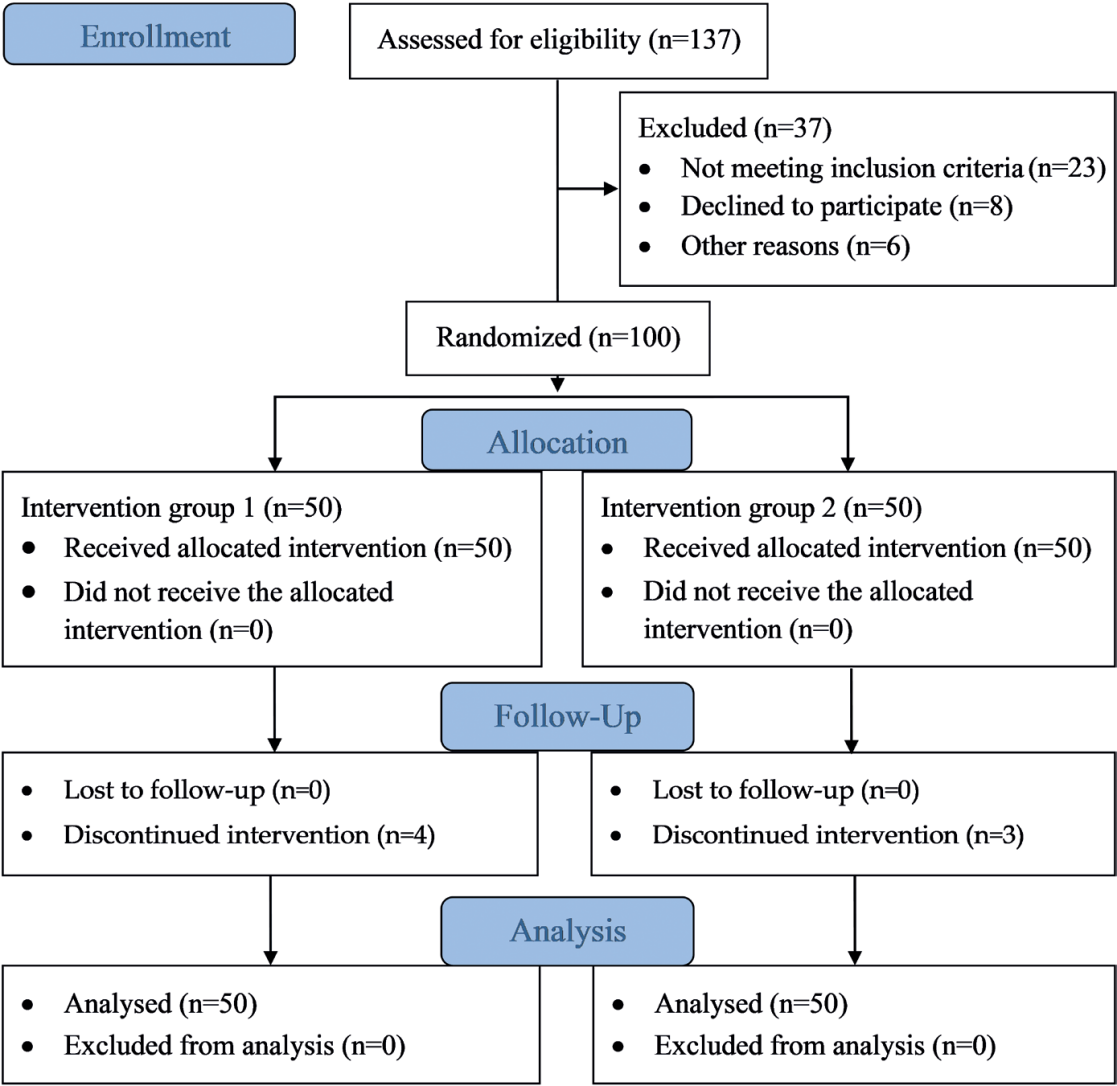
Consolidated Standards of Reporting Trials (CONSORT) (2010) flow diagram showing the study procedures, such as participants’ enrolment, randomization, group allocation, intervention received, follow-up, and analysis.

A total of 137 patients were initially screened for eligibility to participate in the study. Of these, 100 patients met the eligibility criteria and agreed to participate. Subjects were randomly divided into 2 equal groups, with 1 group receiving an accelerated rehabilitation protocol (the experimental group) and the other group receiving a conventional rehabilitation protocol (the control group). Fig. 1 shows a visual representation of the recruitment and retention of patients throughout the study. The demographic characteristics in terms of age, body mass, height, and body mass index (BMI), as well as baseline measurements, are reported in Table I.

**Table I:** Demographic characteristics and baseline measurements of the 2 groups

Variables	Control group (*n* = 50)		Experimental group (*n* = 50)	
	Mean	SD	Mean	SD
Height, m	1.72	0.04	1.72	0.04
Weight, kg	71.13	4.63	71.62	4.68
Body mass index, kg/m^2^	24.13	0.89	24.07	0.90
Age, years	22.09	1.83	21.92	1.74
Pain on 1–10 VAS	9.02	0.82	9.09	0.89
KOOS				
Symptoms	10.96	0.81	11.10	0.81
Pain	11.26	0.88	11.22	0.84
Activities of daily living	0.00	0.00	0.00	0.00
Sports/recreation	0.00	0.00	0.00	0.00
Quality of life	0.00	0.00	0.00	0.00
LSI of Hop test battery				
Vertical jump	0.00	0.00	0.00	0.00
Hop for distance	0.00	0.00	0.00	0.00
Drop jump	0.00	0.00	0.00	0.00
Square hop	0.00	0.00	0.00	0.00
Side hop	0.00	0.00	0.00	0.00

Two-way MANOVA of within- and between-group variations indicated overall significant treatment (Wilks’ lambda = 0.002, F = 6867, and *p* < 0.001) time (Wilks’ lambda < 0.001, F = 125097, and *p* < 0.001), and treatment × time interaction effect (Wilks’ lambda = 0.002, F = 6865, and *p* < 0.001). Furthermore, the time was statistically significant for all outcome measures in favour of subjects receiving an accelerated rehabilitation protocol (*p* < 0.001). The treatment and time*treatment interaction showed statistically significant improvement for all outcome measures in favour of subjects receiving an accelerated rehabilitation protocol (*p* < 0.001), except for pain intensity and activities of daily living (ADL) and pain subscales of the KOOS (*p* > 0.05) **(**Table II).

**Table II:** Baseline, post-intervention, and between-group differences for outcome measures of both groups (control vs experimental)

Variables		Group	Pre-TTT		Post-TTT		Between-subjects effects		
			Mean	SD	Mean	SD	Group	Time	Group time
							*p*-value	*p*-value	*p*-value
KOOS	Symptoms						**< 0.001**	**< 0.001**	**< 0.001**
		Control	10.96	0.81	81.08	0.92			
		Experimental	11.10	0.81	89.52	0.89			
	Pain						0.19	**< 0.001**	0.12
		Control	11.26	0.88	81.86	1.34			
		Experimental	11.22	0.84	82.34	1.55			
	ADL						0.06	**< 0.001**	0.06
		Control	0.00	0.00	9.04	0.83			
		Experimental	0.00	0.00	9.40	1.07			
	Sports/recreation						**< 0.001**	**< 0.001**	**< 0.001**
		Control	0.00	0.00	72.66	1.47			
		Experimental	0.00	0.00	80.88	1.26			
	Quality of life						**< 0.001**	**< 0.001**	**< 0.001**
		Control	0.00	0.00	73.56	1.75			
		Experimental	0.00	0.00	82.00	1.18			
LSI of Hop test battery	Vertical jump						**< 0.001**	**< 0.001**	**< 0.001**
		Control	0.00	0.00	48.30	1.69			
		Experimental	0.00	0.00	72.10	0.93			
	Hop for distance						**< 0.001**	**< 0.001**	**< 0.001**
		Control	0.00	0.00	57.82	1.41			
		Experimental	0.00	0.00	77.94	1.38			
	Drop jump						**< 0.001**	**< 0.001**	**< 0.001**
		Control	0.00	0.00	48.30	1.69			
		Experimental	0.00	0.00	77.00	1.44			
	Square hop						**< 0.001**	**< 0.001**	**< 0.001**
		Control	0.00	0.00	57.66	1.44			
		Experimental	0.00	0.00	80.90	1.28			
	Side hop						**< 0.001**	**< 0.001**	**< 0.001**
		Control	0.00	0.00	51.92	1.34			
		Experimental	0.00	0.00	74.04	1.48			
Pain on 1–10 VAS							0.49	**< 0.001**	0.22
		Control	9.02	0.82	1.66	1.35			
		Experimental	9.09	0.91	1.38	0.95			

**Bold** indicates significant value at *p* ≤ 0.001.

SD: standard deviation; KOOS: Knee injury and Osteoarthritis Outcome Score; LSI: limb symmetry index; ADL: activities of daily living; VAS: visual analogue scale.

For knee effusion, between-group comparisons pre- and post-treatment revealed a statistically insignificant difference (*p* > 0.05). However, the within-group comparison revealed a significant reduction in both groups post-treatment (*p* < 0.001).

## DISCUSSION

ACL rupture is the most common knee injury among athletes, and can result in long-term complications and career-ending conditions (1–4) as 35–45% of injured athletes may not return to perform at their premorbid levels, and recurrent ACL injuries are frequent and can be devastating (15, 16). There is no consensus in the literature on the effectiveness of rehabilitation treatment for ACL rupture or the best protocol to follow for functional outcome improvement (8, 14). The aim of the current study was to fill a gap in the current literature by investigating the functional outcomes of an accelerated rehabilitation protocol vs a conventional rehabilitation protocol for ACLR in amateur athletes. Pain intensity, LSI of the hop test battery, KOOS, and knee effusion were measured as parameters representing functional outcomes, since the current literature lacks objective criteria to determine functional performance (18, 19).

This study found that implementing a 22-week accelerated rehabilitation protocol for amateur athletes after ACLR not only improved knee functional performance regarding all the outcome measures, but was also superior to a conventional rehabilitation protocol. However, they were equally effective in improving pain intensity measured by VAS as well as ADL and pain subscales of the KOOS. The non-superiority of the accelerated protocol compared with the conventional protocol regarding pain reduction could be because the conventional protocol results in substantial pain relief and hence the room for further improvement is small due to the floor effects of the scale. A strength of this study was its randomized, controlled, and single-blinded trial design, which helped minimize bias and increase internal validity. In addition, the study had a relatively large sample size (*n* = 100), which increases the power of the study and improves the generalizability of the results.

The results suggest that both the conventional and accelerated protocols were similarly effective in reducing knee pain intensity and effusion and improving ADL. These results agree with a systematic review, which concluded that a minimally supervised rehabilitation could result in successful ACLR rehabilitation in self-reported knee function, quadriceps, and HT strength (36). In addition, the results of the current study agree with a study that found better self-reported knee function and greater improvement in knee pain, Range of motion (ROM), and thigh muscle circumference in a rehabilitation group (20 weeks) compared with a group with no rehabilitation at all at a 1-year follow-up (37).

Furthermore, the results of the current study are consistent with previous research showing that an accelerated rehabilitation protocol can lead to more rapid and better functional outcomes than a conventional rehabilitation protocol (12, 34, 38, 39). The results suggest that an accelerated rehabilitation protocol may be a more effective treatment option for ACLR in amateur athletes, as it can lead to more rapid recovery and better functional outcomes.

We hypothesize that the superiority of the accelerated rehabilitation protocol compared with the conventional programme could be due to the earlier incorporation of an early progressive resistance exercise regime and dynamic stability exercises, aiming for the early regaining of sufficient neuromuscular control (Appendices S1 and S2). Isometric quadriceps exercises are safe in the first postoperative weeks and improve outcomes after ACLR surgery (7, 38, 39). Open kinetic chain (OKC) quadriceps exercises, when started from week 4 after ACLR with HT, but in a limited ROM between 45° and 90°, could lead to better results (40). The combination of OKC and closed kinetic chain (CKC) quadriceps exercises results in better strength and return to play than CKC exercises alone (14). To optimize outcomes after rehabilitation, neuromuscular training should be added to strength training. Eccentric quadriceps training can be safely incorporated 3 weeks after ACLR and may be the most effective way of restoring quadriceps strength (13, 46). Early regaining of sufficient neuromuscular control, symmetrical motion, and appropriate movement strategies are critical to improving knee function (42, 43).

However, the results of the LSI of the modified single-leg hop test battery and the sports activity and QoL subscales of KOOS revealed that they did not meet the criteria to return to their pre-injury level of sport, despite the significant improvement patients in both groups exhibited in favour of the accelerated rehabilitation group. These results are consistent with a meta-analysis study that found only 38% returned to pre-injury level 2 years after ACLR (44) and a prospective study that found only 23% of patients returned to pre-injury level when applying the LSI of the hop test battery > 90% to all tests and only 10% returned when an LSI of 95% was used (45). Therefore, the accelerated rehabilitation protocol should be extended until patients meet the return to sport criteria, which aligns with the conclusion of the systematic review (7).

Regarding the lack of objective criteria to determine functional performance (18, 19), the fact that 35–45% of injured athletes may not return to perform at their premorbid levels (15, 16), and the fact that recurrent ACL injuries after primary ACLR can be devastating and carry a risk of long-term functional deficits with a rate ranging from 1% to 11% (17), our attempt to quantify functional performance using the LSI of the modified single-leg hop test battery was a successful trial.

### Study limitations

The main limitation of this study was the time needed to recruit adequate participants due to the SARS-CoV-2 (COVID-19) pandemic. Other limitations included the duration of the study; 22 weeks appeared to be insufficient to allow patients to meet their return-to-play criteria. A further limitation is surely that the results apply only to male footballers, thus it is unclear whether the results would be generalizable to professional athletes or individuals with different physical activity levels. In addition, the study did not include a long-term follow-up, hence it is unclear whether the benefits of the accelerated rehabilitation protocol would be sustained over time. Future studies should investigate the long-term effects of an accelerated rehabilitation protocol and whether the results are general by broadening to include women, and athletes other than footballers.

### Conclusion

Application of an accelerated rehabilitation protocol may be a more effective treatment option for ACLR in amateur athletes, as it can lead to more rapid recovery and better functional outcomes compared with a conventional rehabilitation protocol. However, the period of 22 weeks should be extended to allow the achievement of criteria for return to play. Physiotherapists should include an accelerated rehabilitation protocol for faster recovery and better functional outcomes while aiming to treat amateur athletes with ACLR.

## Data Availability

The data-set for the results of this study is available from the corresponding author upon reasonable request.
